# Heavy Atom-Free Triplet Photosensitizers: Molecular Structure Design, Photophysical Properties and Application in Photodynamic Therapy

**DOI:** 10.3390/molecules28052170

**Published:** 2023-02-26

**Authors:** Xiao Xiao, Xiaoyu Zhao, Xi Chen, Jianzhang Zhao

**Affiliations:** State Key Laboratory of Fine Chemicals, Frontiers Science Center for Smart Materials, School of Chemical Engineering, Dalian University of Technology, Dalian 116024, China

**Keywords:** electron transfer, electron spin, fullerene, intersystem crossing, photodynamic therapy, triplet state, radical-enhanced ISC, upper excited states, anti-Kasha’s rule, twisting-induced ISC

## Abstract

Photodynamic therapy (PDT) is a promising method for the treatment of cancer, because of its advantages including a low toxicity, non-drug-resistant character, and targeting capability. From a photochemical aspect, a critical property of triplet photosensitizers (PSs) used for PDT reagents is the intersystem crossing (ISC) efficiency. Conventional PDT reagents are limited to porphyrin compounds. However, these compounds are difficult to prepare, purify, and derivatize. Thus, new molecular structure paradigms are desired to develop novel, efficient, and versatile PDT reagents, especially those contain no heavy atoms, such as Pt or I, etc. Unfortunately, the ISC ability of heavy atom-free organic compounds is usually elusive, and it is difficult to predict the ISC capability of these compounds and design novel heavy atom-free PDT reagents. Herein, from a photophysical perspective, we summarize the recent developments of heavy atom-free triplet PSs, including methods based on radical-enhanced ISC (REISC, facilitated by electron spin–spin interaction), twisted π-conjugation system-induced ISC, the use of fullerene C_60_ as an electron spin converter in antenna-C_60_ dyads, energetically matched S_1_/T_n_ states-enhanced ISC, etc. The application of these compounds in PDT is also briefly introduced. Most of the presented examples are the works of our research group.

## 1. Introduction

Photodynamic therapy (PDT) has attracted significant attention in recent years since it is promising as a new method for the treatment of cancer [1,2,3,4,5,6,7]. The principal mechanism involves the molecular oxygen (O_2_) in the tumor tissue being sensitized to singlet oxygen (^1^O_2_), a potent oxidizing reagent, which causes cell death, by using a triplet photosensitizer (PS) and light irradiation. This is the so-called type II PDT. Type I PDT is related to the production of a superoxide radical anions. This topic will not be discussed in this review [7,8]. The underlining photophysical process of the type II PDT is that upon photoexcitation, first the singlet excited state of the PS is populated, e.g., via the S_0_→S_1_ transition, then the triplet excited state (e.g., T_1_) of the PS is populated, via the intersystem crossing (ISC) process, e.g., S_1_→T_1_ or S_1_→T_n_→T_1_. Subsequently, ^1^O_2_ is produced through intermolecular triplet energy transfer from the PS molecules to molecular oxygen (which is in a triplet ground state) in the tumor tissue. This potent oxidizing reagent causes cell death. The advantages of PDT, compared to the conventional chemotherapy, include the low toxicity of PDT reagents (compared with conventional chemotherapy reagents), non-resistant features, and the targeted character [7]. Although many factors have to be taken into account for a successful clinical PDT reagent, the light absorptivity and ISC efficiency are critical, from a photochemical viewpoint. Conventional PDT reagents are limited to porphyrin compounds, though these compounds are usually difficult to synthesize, and their purification and derivatization are also challenging [9]. Therefore, it is highly desired to develop new triplet PSs based on a new paradigm of molecular structures. However, this is not a trivial task from a photochemical point of view, because the ISC of heavy atom-free organic compounds is elusive and difficult to predict, and molecular structure characters ensuring efficient ISC in heavy atom-free triplet PSs are not well understood [10,11,12]. Great efforts also have been devoted to the theoretical study the ISC in heavy atom-free organic compounds [13,14,15]. Concerning this aspect, charge recombination-induced ISC in electron donor-acceptor compounds has been studied and summarized recently [6,16,17,18], as well as the ISC enhanced by thionation of carbonyl groups in the PS molecules [19,20,21]. In this review article, we will introduce the recent development of heavy atom-free triplet PSs that show strong absorption of visible light and efficient ISC. We will focus on the molecular structure design and related ISC mechanisms, and the application of several reagents in the PDT study will be briefly introduced.

## 2. Radical-Enhanced ISC (REISC)

### 2.1. REISC Based on Bodipy-TEMPO Dyads

**Figure 1:**
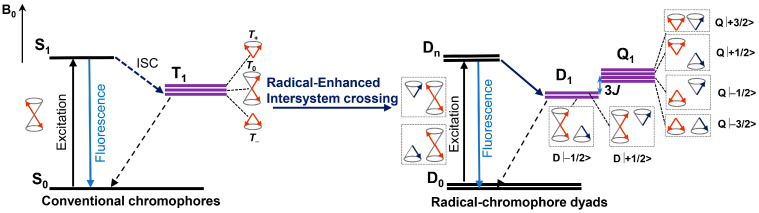
The mechanism of REISC in the chromophore-radical dyads. D_0_: doublet ground state, D_n_: excited doublet state, D_1_: the lowest excited doublet state, Q_1_: the lowest excited quartet state, 3*J*: energy splitting (*J* < 0), T_+_, T_0_, T_−_, D|+1/2>, D|−1/2>, Q|+3/2>, Q|+1/2>, Q|−1/2>, Q|−3/2>: sublevel, B_0_: magnetic field. Reproduced with permission from reference [28], copyright 2021, American Chemical Society.

It is known that fluorescence of an organic chromophore can be quenched by persistent radicals, such as 2,2,6,6-tetramethylpiperidinyloxyl (TEMPO), either by covalent bond connection to the fluorophore or intermolecular collision [22]. The assumption is that a radical is a paramagnetic species, and the singlet excited state (i.e., the emissive state) of an organic molecule can be quenched by it; in other words, the radical enhances the non-radiative decay of the emissive S_1_ state. However, in most cases, a detailed mechanism underlining the fluorescence quenching has not been unraveled [23,24]. In fact, with the attachment of a stable radical to a chromophore (this part is for light absorption), a three-spin system is formed upon photoexcitation of the chromophore part. The electron spin dynamics of these molecular systems have been studied well with pulsed laser excited time-resolved electron paramagnetic resonance (TREPR) spectroscopy, mainly within the field of electron spin chemistry [22,25]. Given that the electron spin carrier of the radical is close to the chromophore, the ground state of the dyad is a doublet state (D_0_). Upon photoexcitation, first the singlet excited state of the chromophore is populated, in which the chromophore is in a closed-shell electronic configuration. The overall spin multiplicity is a doublet excited state (D_n_) based on the electron spin–spin exchange interaction in this three-electron spin system. Then, the system may relax to the low-lying doublet excited state (D_1_ state), in which the chromophore is in an open-shell electronic configuration, but the electrons adopt such a spin coupling that the chromophore itself has triplet features, although the overall spin multiplicity of the three-electron system is a doublet state (D_1_). Note that in this case, the ISC of the chromophore becomes electron spin allowed, i.e., D_n_→ D_1_, which is contrary to the S_1_→ T_1_ of the chromophores in the absence of the radical (Figure 1). This is the mechanism of radical-enhanced ISC (REISC) [25,26,27,28]. Previously studies in this area focused on the electron spin dynamics by using the pulsed laser excited TREPR spectra, such as the detection of the quartet state, or the electron spin polarization of a radical, as a result of the spin–spin interaction with the triplet excited state. However, no study was performed to explore the possibility of designing a heavy atom-free triplet PS based on REISC.

**Figure 2:**
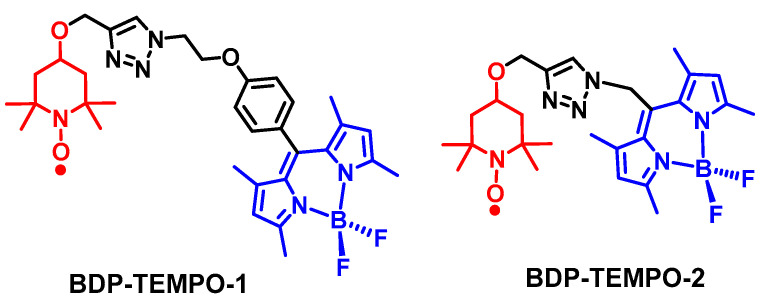
Molecular structures of chromophore-radical dyads containing a visible light-harvesting chromophore and a stable radical, BDP-TEMPO-1 and BDP-TEMPO-2.

Recently, we demonstrated that the REISC effect can be used to design efficient heavy atom-free triplet PSs [29]. We selected a popular chromophore, the dipyrromethane (Bodipy), as the visible light-harvesting unit, because this kind of chromophore shows strong visible light-harvesting ability, good stability, feasible derivatization [30,31,32,33], etc. We selected TEMPO as the radical (electron spin carrier). The two units are readily connected by a click reaction. We used linkers with different lengths to connect the Bodipy chromophore and the TEMPO radical (BDP-TEMPO-1 and BDP-TEMPO-2, Figure 2). The rational is that, with a shorter linker, the electron spin–spin interaction between the triplet excited state and the radical will be strong. This will lead to an efficient D_n_→D_1_ transition, i.e., the ISC of Bodipy will be efficient and the T_1_ state of the Bodipy unit will be produced with a high efficiency; however, the T_1_ state lifetime will probably be substantially shortened, due to the D_1_→D_0_ transition [34]. This is detrimental from the point of view of triplet PS, because the sensitizing of ^1^O_2_ is an intermolecular and diffusion-controlled process, ao a longer triplet state lifetime is beneficial for the intermolecular energy or electron transfer processes [35,36,37]. Therefore, we also used a relatively longer linker in the Bodipy-TEMPO dyad, with the attempt to fine tune the electron spin–spin interaction magnitude between the chromophore and the TEMPO, so that the D_n_→D_1_ is efficient (i.e., for the Bodipy chromophore, it is S_1_→T_1_), whereas the D_1_→D_0_ is inhibited to some extent, so that the triplet state lifetime of the chromophore will be prolonged. It has been shown previously that with a shorter distance between the radical and the chromophore (such as perylene), the triplet state of the chromophore is greatly shortened in some cases [34]. With a longer distance, the triplet state lifetime is longer [27]. Note that with the normal optical transient absorption spectra, the D_1_ and Q states of the chromophore-radical dyads cannot be discriminated directly, because the difference of these states are only the electron spin configuration, not the molecular orbital occupancy. It is different from the S_1_/T_1_ states confined on a chromophore. However, the D_1_ and Q states can be well discriminated by pulsed laser excited TREPR spectra [27].

Both dyads (BDP-TEMPO-1 and BDP-TEMPO-2) show similar UV–vis absorption maxima at 504 nm and 523 nm, respectively, which is the featured absorption of the respective Bodipy unit, indicating negligible electronic interaction between the radical and the Bodipy chromophore in the dyads at ground state (the different absorption wavelengths of the two dyads are due to the different linkers between the Bodipy chromophore and the TEMPO units, confirmed by the reference compounds containing no TEMPO unit). The fluorescence quantum yields (Φ_F_) of BDP-TEMPO-1 and BDP-TEMPO-2 were determined as 29% and 5.0%, respectively, indicating the quenching of the fluorescence of the Bodipy unit by attachment of the radical unit. As an approximation of the ISC efficiency, the singlet oxygen quantum yields (Φ_Δ_, determined with a ^1^O_2_ scavenger, 1,3-diphenylisobenzofuran (DPBF)) of the two dyads were determined as 14% and 56%, respectively. The Φ_Δ_ of the native Bodipy is negligible. These results infer that the electron spin–spin interaction in BDP-TEMPO-2 is stronger than that in BDP-TEMPO-1, because of the shorter linker in BDP-TEMPO-2. Nanosecond transient absorption (ns-TA) spectra show that the triplet state confined on the Bodipy unit was populated upon pulsed laser excitation, the triplet state lifetimes of the BDP-TEMPO-1 and BDP-TEMPO-2 are 190 μs and 62 μs, respectively [29]. Note that all the triplet state lifetimes are measured in deaerated solution. This is a reasonable result, because of the electron spin–spin interaction magnitude between the triplet chromophore and the radical; thus, the D_1_→D_0_ relaxation kinetics is dependent on the linker length. Although the triplet state lifetime of BDP-TEMPO-1 is longer, the ISC quantum yield is lower than BDP-TEMPO-2. Concerning the design of heavy atom-free triplet PSs, BDP-TEMPO-2 represents a balanced REISC effect, and both decent ISC yield and triplet state lifetime are attained [3,4,11,12]. To the best of our knowledge, this is the first example with a clear purpose of designing a heavy atom-free triplet PS with the REISC effect [11,12,38].

The electron spin dynamics of the dyad BDP-TEMPO-2 were studied with TREPR spectra. In frozen solution at 80 K, two triplet states, spin coupled to the radical, were observed. With spectral simulation, the electron exchange integral for the radical and the triplet state of the Bodipy chromophore was observed as 170 MHz and 1.2 × 10^4^ MHz, respectively. These two situations are attributed to the extended and folded geometry of the dyad, which is in agreement with the molecular dynamic simulations. The TREPR spectra of the dyad in fluid solution at room temperature were also studied. Firstly, an absorptive TREPR signal was observed, which later evolved into an emissive signal; this electron spin polarization transition is attributed to the radical triplet pair mechanism [29].

### 2.2. REISC Based on NDI-TEMPO Dyads

**Figure 3:**
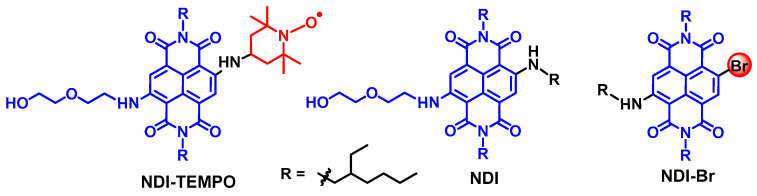
Molecular structures of the chromophore-radical dyad NDI-TEMPO. The molecular structures of the reference compounds NDI and NDI-Br are also presented.

This molecular structure paradigm for the design of heavy atom-free triplet PSs with predictable ISC capability is versatile and it can be feasibly extended to other chromophores, such as the cyanine dyes [39,40]. Concerning their application in PDT, absorption in the long wavelength spectral range is desired, because of their large penetration depth in tissues. Thus, we prepared a naphthalenediimide (NDI)-TEMPO dyad (Figure 3), which absorbs in the red spectral range (the absorption band is centered at 606 nm, with a molar absorption coefficient of *ε* = 2 × 10^4^ M^−1^ cm^−1^) [41]. This red-shifted absorption wavelength, as compared to the native NDI chromophore, is attributed to the amino substitution on the NDI chromophore [42,43,44].

**Figure 4:**
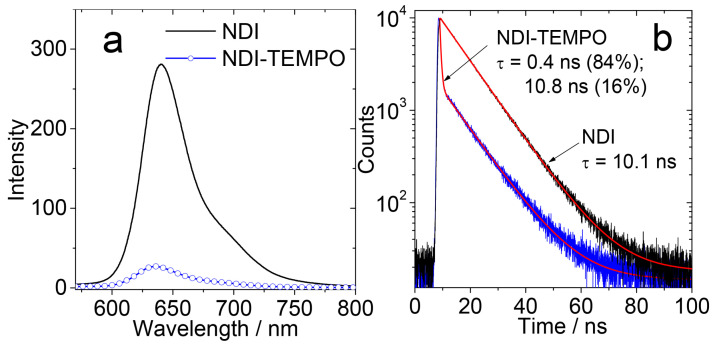
(**a**) Fluorescence emission spectra of NDI and NDI-TEMPO in toluene, *λ*_ex_ = 560 nm. Optically matched solutions were used, *c* ≈ 1.0 × 10^−5^ M. (**b**) Fluorescence decay curves of NDI and NDI-TEMPO in toluene. *c* = 1.0 × 10^−5^ M, *λ*_ex_ = 635 nm, 20 °C. Reproduced with permission from reference [41], copyright 2018, Wiley-VCH.

As compared to the decent Φ_F_ of the reference compound NDI (24%), the fluorescence of NDI-TEMPO is much weaker (3%; Figure 4a). Conversely, the Φ_Δ_ increased from NDI (Φ_Δ_ = 2%) to Φ_Δ_ = 50% for NDI-TEMPO. Thus, this is an interesting example that the non-radiative relaxation of a chromophore was transformed to ISC, with the attachment of a radical. For the reference compound NDI-Br, the Φ_Δ_ is 57%. These results show that the enhancement of the S_1_→T_1_ ISC of the chromophore by either the heavy atom effect of Br atom or by the REISC effect of the TEMPO radical can be similar. We observed an interesting fluorescence lifetime change upon the attachment of radical (Figure 4b). The reference chromophore NDI gives a fluorescence lifetime of 10.1 ns (single exponential decay). For NDI-TEMPO, however, a distinct biexponential decay with lifetime of 0.4 ns (84%)/10.8 ns (16%) was observed.

**Figure 5:**
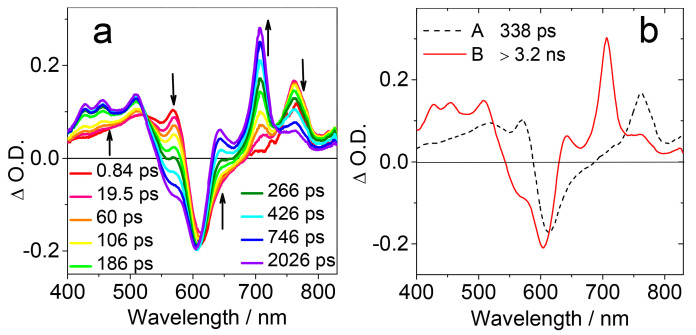
(**a**) Femtosecond transient absorption spectra of NDI-TEMPO at different delay times (*λ*_ex_ = 610 nm). (**b**) Evolution associated difference spectra (EADS) of NDI-TEMPO obtained by global fitting and target analysis of the fs-TA spectral data. *c* = 2.5 × 10^−4^ M in toluene, 20 °C. Reproduced with permission from reference [41], copyright 2018, Wiley-VCH.

In order to characterize the ISC kinetics, facilitated by the electron spin–spin interaction between the photoexcited chromophore and the TEMPO radical, the femtosecond transient absorption (fs-TA) spectra of NDI-TEMPO were recorded (Figure 5a). Upon fs laser excitation, excited state absorption (ESA) bands centered at 760 nm and in the range of 400–580 nm were observed, which are attributed to the S_1_ state of the amino-substituted NDI chromophore. A ground state bleaching (GSB) band centered at 612 nm was observed. With the increase of the delay time, the ESA bands decreased, and new ESA bands in the range of 400–500 nm, as well as at 700 nm, developed. These ESA bands are assigned to the triplet excited state of the amino-substituted NDI chromophore and this conclusion is supported by the ns-TA spectra.

In order to explicitly unravel the species involved in the photophysical processes, global fitting and target analysis of the fs-TA spectral data were performed, based on a sequential kinetic model (Figure 5b). The first species has a lifetime of 338 ps, which is assigned to the S_1_ state of the amino-substituted NDI chromophore. The second species gives absorption profile similar to the T_1_ state of the amino-substituted NDI chromophore; thus, it is assigned to the T_1_ state of the chromophore. The lifetime of this state is beyond the detection time window of the fs-TA spectrometer.

**Figure 6:**
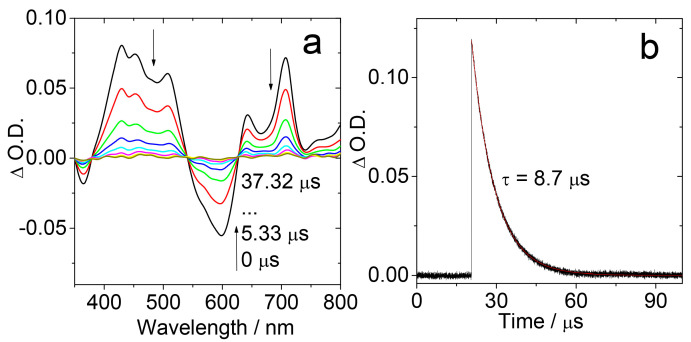
(**a**) Nanosecond transient absorption spectra of NDI-TEMPO (*λ*_ex_ = 608 nm). (**b**) Decay trace monitored at 430 nm (black line: experimental spectrum, red line: fitting result). *c* = 5.0 × 10^−6^ M in toluene, 20 °C. Reproduced with permission from reference [41], copyright 2018, Wiley-VCH.

In order to obtain the triplet state information of the NDI-TEMPO, the ns-TA spectra of the compound were recorded (Figure 6a). ESA bands in the range of 400–500 nm and 650–750 nm were observed. Moreover, a GSB band centered at 600 nm was also observed. These bands decay monotonically with the extension of the delay time after laser irradiation and the triplet state lifetime was determined as 8.7 μs (Figure 6b). Note that the doublet state, the quartet states, and the triplet state of the NDI chromophore cannot be discriminated by ordinary optical spectral methods, but rather by TREPR spectra [45,46,47].

The TREPR spectra of NDI-TEMPO in fluid solution at room temperature were studied. Initially broad, emissive spectra were observed, and at a longer delay time (3.0 μs), an absorptive signal of the TEMPO unit was observed. This is opposite to the result of BDP-TEMPO-2 [29]. These results demonstrated the rich electron spin chemistry of the REISC molecular systems [27].

## 3. Twisted π-Conjugation-Induced ISC in Aromatic Compounds

For aromatic compounds, which generally have planar molecular structures, the ISC is usually poor [10]. This is due to the large S_1_/T_1_ states energy gap of these compounds (given that the two states share the same electronic configuration), which results from the large electron exchange energy (*J*) of the two electrons in the frontier molecular orbitals (the S_1_/T_1_ state energy gap is 2*J*). In particular, some planar aromatic compounds have a quite strong ISC due to the closely lying S_1_/T_n_ states (such as anthracene and tetracene), or the ^1^(n,π*) to ^3^(π,π*) transition (such as phenalenone). Moreover, the planar molecular structure will cancel the spin–orbit coupling effect, which makes the spin–orbit coupling (SOC) matrix elements negligible [48]. Thus, ISC is rare for aromatic organic compounds with a large, planar, π-conjugated framework. It is known that helicenes, i.e., hydrocarbons with highly twisted molecular structures, show efficient ISC [49,50]. It was proposed that the twisted π-conjugation system induces the SOC effect; thus, the ISC is enhanced in these heavy atom-free organic molecules [48]. We envision that this property may be exploited as a novel molecular structure paradigm for designing new, heavy atom-free triplet PSs. However, it is clear that helicenes are most likely not the ideal candidates for this purpose, because they are difficult to derivatize and the native helicenes usually show absorption in the UV or blue spectral region.

**Figure 7:**
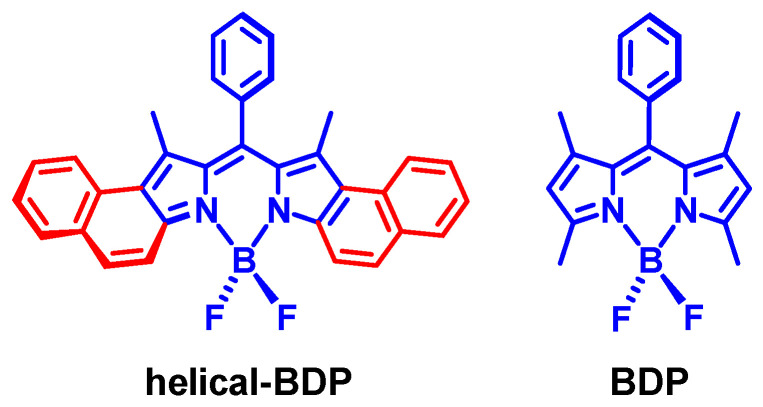
Molecular structures of a twisted Bodipy derivative showing efficient ISC (helical-BDP) and a typical native Bodipy compound (BDP).

**Figure 8:**
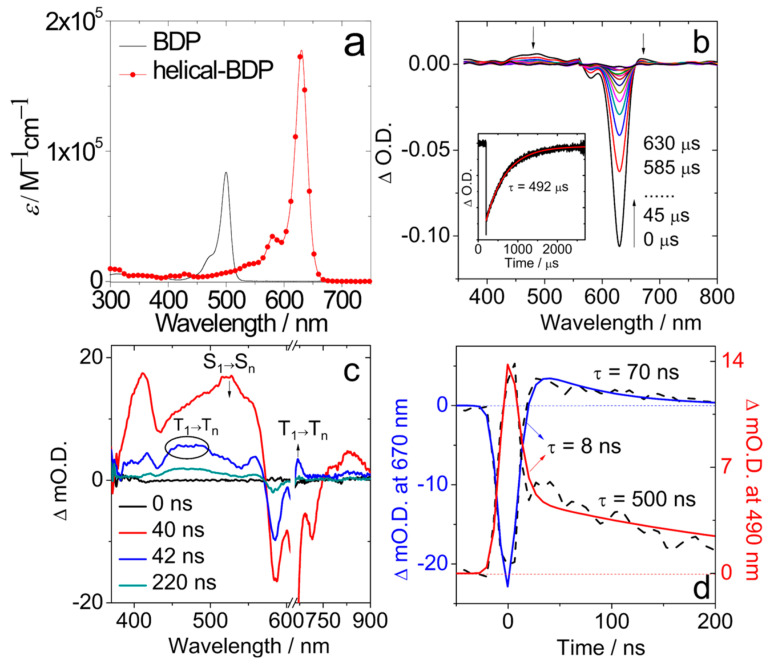
(**a**) Molar absorption coefficient of helical-BDP in DCM. (**b**) ns-TA spectra of helical-BDP (*λ*_ex_ = 628 nm, *c* = 1.25 × 10^−6^ M). Inset: Decay trace of helical-BDP at 610 nm, *c* = 3.1 × 10^−7^ M. 20 °C. (**c**) Sub-nanosecond TA spectra of helical-BDP in toluene (λ_ex_ = 640 nm) at 0 ns, 40 ns, 42 ns, and 220 ns. (**d**) Kinetic traces at 670 nm and 490 nm showing the time constant for triplet state formation (black dash line: experimental spectrum, blue and red solid lines: fitting results). Reproduced with permission from reference [54], copyright 2020, Wiley-VCH.

Previously, twisted, fused perylenebisimide (PBI) dimers were reported showing decent ISC capability [51,52]. However, the derivatization of the compound is not feasible and, indeed, further modification of the twisted PBI dimers was not reported. We noted an interesting Bodipy derivative, helical-BDP, with a slightly twisted molecular structure, showing very weak fluorescence (Figure 7) [53]. This is strange because Bodipy compounds are known for exhibiting strong fluorescence (non-radiative decay is greatly inhibited for this chromophore) [12]. Moreover, we noted that helical-BDP shows a strong absorption of visible light at 630 nm (Figure 8a), inferring that the S_0_→S_1_ transition is allowed, as is the S_1_→S_0_ transition; therefore, there must be another non-radiative decay channel competing with the S_1_→S_0_ decay that finally quenches the fluorescence of this heavy atom-free Bodipy derivative. One of the possible non-radiative decay channels of the otherwise emissive S_1_ state is ISC. Therefore, we studied the ISC of helical-BDP in detail [54]. The Φ_Δ_ value of the compound was determined as 36%; this is an exceptional result compared to the negligible Φ_Δ_ of the native Bodipy [11,12,32,33].

The ISC capability of helical-BDP was unambiguously confirmed by ns-TA spectra, which show weak ESA bands in the range of 400–600 nm, and a strong GSB band centered at 649 nm (Figure 8b). The triplet state lifetime was determined as 492 μs in fluid solution at room temperature. This is much longer than that of the iodinated Bodipy (ca. 276 μs), for which the ISC is resulted with the heavy atom effect [55]. With sub-nanosecond transient absorption spectra, it was found that the ISC takes ca. 8 ns (Figure 8c and 8d), which is rather slower than the ISC of the iodinated Bodipy (131 ps) [56].

Concerning the normal optical spectral study of ISC, the ISC rate constant was determined, or the ISC yields could be obtained by comparison of the ISC rate constant and the relaxation of the S_1_ state of the native chromophore which does not show ISC. However, some critical information is missing in these optical spectral characterizations, i.e., the electron spin selectivity of the ISC, or the non-Boltzmann population of the three sublevels of the T_1_ state, i.e., T_x_, T_y_, and T_z_ [57,58,59]. Due to the anisotropic SOC constants, the population of the three sublevels severely deviates from the Boltzmann distribution, i.e., the electron spin is highly polarized [60,61,62]. However, this information is usually not manifested in ordinary optical spectroscopies. Additionally, the energy gap between the T_x_, T_y_, and T_z_ is <1 cm^−1^ for organic compounds; thus, discrimination of the three sublevels is impossible with normal optical spectroscopic methods. Concerning this aspect, pulsed laser excited TREPR spectroscopy is very useful [57,58,59,63]. The polarization of the electron spin of the triplet state can be directly characterized with the TREPR spectra, manifested by the enhanced absorptive bands or the emissive bands in the TREPR spectra, especially at the six turning points (corresponding to the transitions of the molecules at canonical orientation against the external magnetic field) of the triplet state TREPR spectra of randomly oriented molecules in the magnetic field of the EPR spectrometer [57,58,59,63]. Moreover, the zero-field splitting (ZFS) parameters *D* and *E* can be obtained by simulation of the triplet TREPR spectra. These are fundamental parameters of the triplet state [64], and they quantitatively characterize the electron spin–spin dipolar interaction of the two electrons of the triplet state, and the rhombicity of the triplet state wavefunction, respectively [59]. 

Previously, we determined the ZFS *D* and *E* parameters of diiodoBodipy as −105.7 mT and 23.4 mT, respectively [65]. The negative *D* value of the triplet state of Bodipy shows that the ZFS *Z* axis lies within the plane of the molecule, and the spatial distribution of the triplet state wavelength is prolate in shape [59]. We also found that the Bodipy chromophore without the iodo substitution has a larger *D* (−156.3 mT) and *E* value (36.4 mT) [66]. The methyl groups at the 1,3,5,7-positions impose a significant effect on the magnitude of the *D* and *E* values of the triplet state of Bodipy. The magnitude of the *D* value indicates the delocalization of the triplet state wavefunction. With a large delocalization, the dipolar interaction will be weaker and smaller *D* values will be obtained. It would be interesting to characterize the triplet state wave function distribution of helical-BDP.

**Figure 9:**
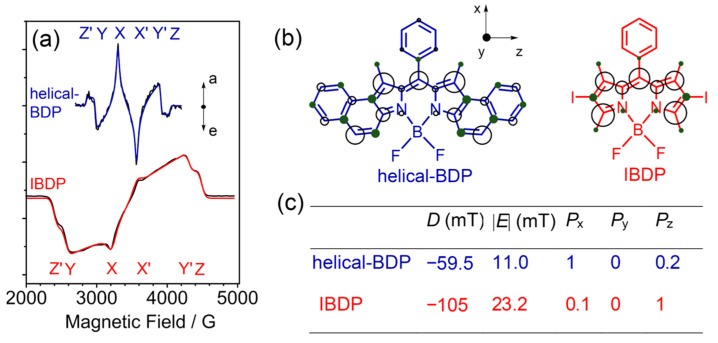
(**a**) TREPR spectra of helical-BDP (*λ*_ex_ = 555 nm) and IBDP (*λ*_ex_ = 532 nm) in toluene/MeTHF (*v*/*v*, 3:1) frozen mixed solution (black line: experimental spectrum, blue and red lines: simulation results), *c* = 3.0 × 10^−5^ M, 80 K. (**b**) Spin density distribution of helical-BDP and IBDP. (**c**) Parameters used for the simulations of the spectra in (**a**). Reproduced with permission from reference [54], copyright 2020, Wiley-VCH.

The TREPR spectrum of the triplet state of the helical-BDP was recorded (Figure 9). It is clear that the TREPR spectrum of helical-BDP is much narrower than that of the native Bodipy, which infers a smaller ZFS *D* parameter for the triplet state of helical-BDP than the triplet state of native Bodipy. Simulation of the TREPR spectrum gives the ZFS *D* parameter as −59.5 mT, which is much smaller than that of the triple state of the native Bodipy (IBDP), being −105 mT. These results indicates that the electron spin dipolar interaction in the triplet state of helical-BDP is much weaker than that in native Bodipy; in other words, the triplet excited state wavefunction of helical-BDP is more delocalized than that of the native Bodipy. The ZFS *E* parameter of helical-BDP is only half that of the native Bodipy, indicating that the rhombicity of the spatial distribution of the triplet state wavefunction is less significant than the native Bodipy.

Another striking feature of the triplet state TREPR spectrum of helical-BDP is the electron spin polarization (ESP) phase pattern. A (a, e, a, e, a, e) polarization was observed, whereas for the triplet state of the native Bodipy, it is (e, e, e, a, a, a), this is the typical ESP phase pattern of the triplet state TREPR spectra of organic chromophores, for which the triplet excited states are accesses with the SOC effect. The results of helical-BDP show that the population ratio of the three sublevels of the T_1_ state of helical-BDP is drastically different from that of the native Bodipy. This is confirmed by the simulation results of the experimental TREPR spectra. Our results show that the electron spin chemistry of the ISC of organic compounds is richer than previously thought [57,58,59,63]. 

Helical-BDP contains no heavy atoms, the ISC quantum yield is decent, and the red fluorescence is moderate (quantum yield: 21%); thus, it was used as a novel PDT reagent [54]. Since it is hydrophobic, it has been used to prepare nanoparticles, and an in vivo PDT study with an artificial metastatic tumor shows antitumor immunity amplification at an ultra-low dose (0.25 μg kg^−1^), which is several hundred times more potent than the existing PDT reagents (>1.4 mg kg^−1^) [54]. These results show that the chromophores with twisted molecular structures can be developed as a novel type of heavy atom-free triplet PSs for PDT.

**Figure 10:**
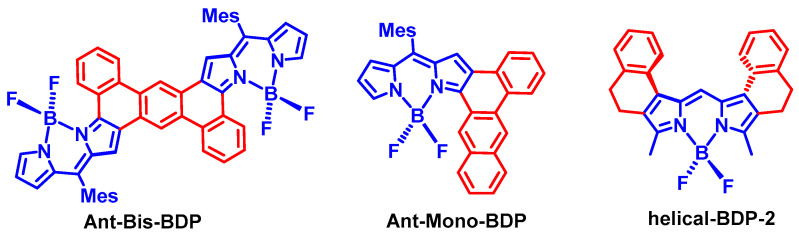
Bodipy derivatives with a twisted π-conjugation system in the molecular structure. ISC was observed for these compounds.

Jun Kawamata and Taku Hasobe et al. studied a series of twisted Bodipy compounds (Ant-Bis-BDP and Ant-Mono-BDP; Figure 10), and moderate ISC quantum yields of 3.5–42% were observed [67]. We also studied other Bodipy derivative, helical-BDP-2 (Figure 10) [68,69]. The Φ_F_ of helical-BDP-2 is ca. 30%, and the ISC quantum yield is ca. 60%. A triplet state lifetime of 198 μs was observed using ns-TA spectra. Interestingly, the triplet state TREPR spectrum of helical-BDP-2 shows an ESP phase pattern of (a, a, e, a, e, e), which is different from that of both helical-BDP and the native Bodipy. Simulation of the TREPR spectrum of the compound gives a ZFS *D* parameter of −69.5 mT. In comparison, the ZFS *D* parameter of 2,6-diiodoBodipy is −104.6 mT. The population ratio of the three sublevels of the T_1_ state is drastically different from that of 2,6-diiodoBodipy (IBDP) and helical-BDP [69].

**Figure 11:**
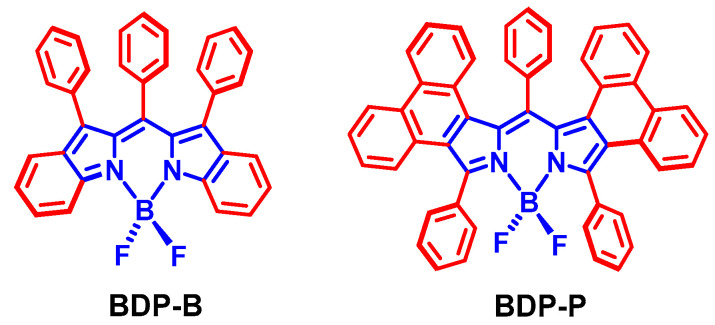
Bodipy derivatives showing that the ISC quantum yields are not directly correlated with the torsion of the π-conjugation system.

Recently we found that a relatively simple PBI derivative also shows high ISC [52,70,71]. Thus, we envision that this strategy can be extended to other chromophores, to design heavy atom-free triplet PSs [11,12]. However, one should be careful when using this strategy, because our recent results show that the ISC quantum yields of the twisted Bodipy compounds are not directly dependent on the torsion of the π-conjugation system (Figure 11) [72]. For instance, the π-conjugation system of BDP-B is only slightly distorted, and it is highly distorted for BDP-P. However, we found a decent ISC quantum yield for BDP-B (up to 29%), yet the ISC of BDP-P is negligible. Moreover, a dark state was identified for BDP-B, which is unusual for Bodipy derivatives [33]. 

## 4. Fullerene C_60_ as an Electron Spin Converter in the Design of Heavy Atom-Free Triplet PSs

### 4.1. Heavy Atom-Free Triplet PSs Based on Bodipy-C_60_ Dyads

A molecular structure paradigm for heavy atom-free triplet PSs that can be feasibly extended to different chromophore is highly desired, because this will make the new triplet PSs readily available. REISC, introduced in the previous section of this manuscript, is one of such examples. We believe that another method is to use the efficient ISC of fullerenes, such as C_60_ [73]. The ISC of C_60_ has been known for decades, but it was never exploited for any photochemical applications. C_60_ itself is not an ideal triplet PS, because of its very poor solubility in ordinary organic solvents, especially in aqueous solution. Moreover, the visible light absorptivity of C_60_ is poor. Although C_60_ has been used in PDT studies [73,74], new strategies should be developed to fully exploit the potential of the ISC capability of C_60_.

**Figure 12:**
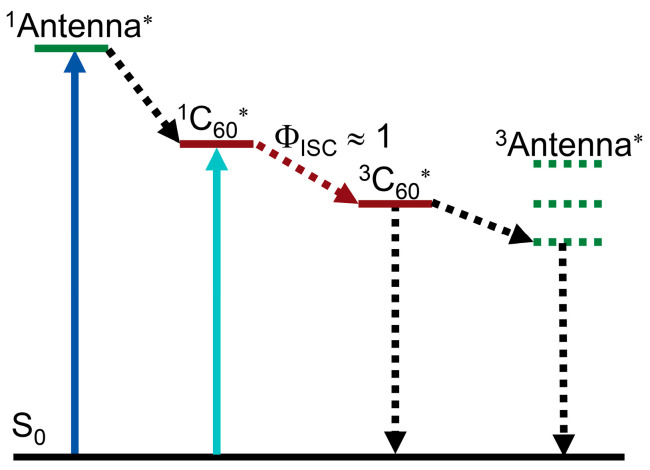
Mechanism for using of C_60_ as an electron spin converter to prepare heavy atom-free triplet PSs (antenna-C_60_ dyads). * stands for the excited state.

We noted that in some Bodipy-C_60_ dyads, ISC was observed [75,76]. In the case of a light-harvesting chromophore (i.e., antenna) with proper S_1_ state energy attached to C_60_, we envisage that Föster resonance energy transfer (FRET) may occur, given that the S_1_ state energy of the antenna is higher than the S_1_ state of C_60_ (1.72 eV) [73]. Upon photoexcitation of the antenna and subsequent FRET to C_60_, efficient ISC of C_60_ will occur, resulting in a long-lived triplet state (the intrinsic triplet state lifetime of C_60_ is ca. 40 μs) [73]. The final localization of the triplet state is dependent on the relative T_1_ state energy order of the antenna and the C_60_ unit (Figure 12). This antenna-C_60_ dyad has the advantage of the complementary visible light-harvesting capability of the antenna, and the efficient ISC of the C_60_ part, for construction of a heavy atom-free triplet PS showing both strong absorption of visible light and efficient ISC. This could be an interesting heavy atom-free triplet PS molecular paradigm that can be easily extend to different chromophores, as long as intramolecular electron transfer is suppressed [77], and the antenna possesses proper excited state energy.

**Figure 13:**
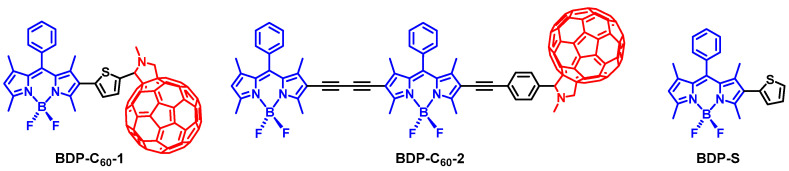
Molecular structures of the antenna-C_60_ heavy atom-free triplet PSs, BDP-C_60_-1 and BDP-C_60_-2. BDP-S is a reference compound.

**Figure 14:**
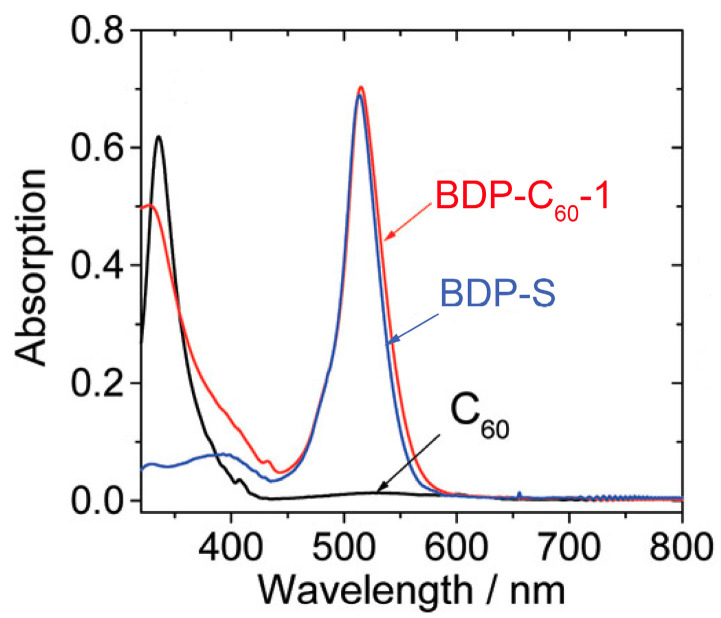
UV−vis absorption spectra of BDP-C_60_-1 and the antennas of the dyads BDP-S. *c* = 1.0 × 10^−5^ M in toluene, 20 °C. Reproduced with permission from reference [78], copyright 2012, American Chemical Society.

In order to test this molecular design rational, we prepared two antenna-C_60_ dyads (Figure 13) [78], in which different antenna units can be readily induced by the Prato reaction. The purpose of using different antenna units is to show the readily changeable absorption wavelength, as well as the possibility of further derivatization, such as attaching a targeting moiety for PDT studies [4]. UV–vis absorption of the dyad BDP-C_60_-1 shows an intense absorption band at 515 nm (Figure 14), which is attributed to the antenna, because the C_60_ unit gives a very weak absorption at the same wavelength. These results show that there is no electronic interaction between the C_60_ and the Bodipy units at the ground state (S_0_ state). Similar results were observed for BDP-C_60_-2 [78]. The energy of the ^1^BDP* state (ca. 2.46 eV) is higher than that of ^1^C_60_* state (1.72 eV) [65,73]; thus, the FRET from the ^1^BDP* state to the ^1^C_60_* state can occur. The fluorescence spectra show that the emission of the antenna is quenched completely, indicating either electron transfer or FRET to the C_60_ unit. 

**Figure 15:**
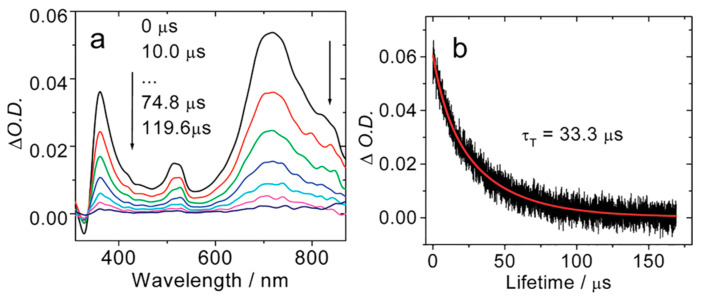
(**a**) Nanosecond transient absorption spectra of BDP-C_60_-1 (*λ*_ex_ = 532 nm). (**b**) Decay traces of the transient absorption of BDP-C_60_-1 at 720 nm (black line: experimental spectrum, red line: fitting result). In deaerated toluene, 25 °C. Reproduced with permission from reference [78], copyright 2012, American Chemical Society.

In order to study the triplet excited state of the C_60_-BDP dyads, the ns-TA spectra of the compounds upon nanosecond pulsed laser excitation were recorded (Figure 15). For BDP-C_60_-1, positive absorption bands centered at 720 nm, 510 nm, and 370 nm were observed. The absorption band centered at 720 nm is the characteristic absorption of the ^3^C_60_* state [79], which is supported by the bleaching band at ca. 360 nm. No bleaching band at ca. 510 nm was observed. The energy of the ^3^BDP* state (1.65 eV) is higher than that of the ^3^C_60_* state (1.50 eV) [80,81]. Based on these spectral data, we concluded that the T_1_ state of the dyad is localized on the C_60_ unit in the dyad, not on the Bodipy unit. The triplet state lifetime was determined as 33.3 μs, which is typical for the ^3^C_60_* state [73]. Similar results were observed for BDP-C_60_-2. The dyads were used as triplet PSs for triplet–triplet annihilation upconversion, and an upconversion quantum yield of 7% was observed [78]. These results demonstrate that this molecular structure profile is a promising paradigm for the design of heavy atom-free triplet PSs. This strategy is feasibly extendable to other fullerenes, for instance, C_70_ [82].

### 4.2. Heavy Atom-Free Triplet PSs Based on StyrylBodipy-C_60_ Dyads

**Figure 16:**
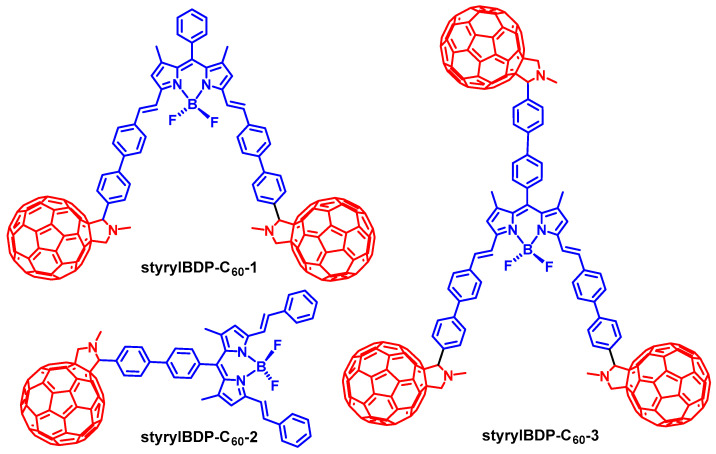
Molecular structures of the antenna-C_60_ heavy atom-free triplet PSs based on red light-absorbing styrylBodipy antennae.

The localization of the triplet state in the antenna-C_60_ dyads (i.e., the triplet state can be located on the antenna or C_60_ unit) is also important, because the triplet excited state redox potential and lifetime impose substantial impact on the application performance of the dyads in PDT or photoredox catalysis. The localization of the triplet state of the dyads can be readily tuned by using antenna having different excited state energy. We demonstrated this idea with the preparation of styrylBodipy-C_60_ dyads (Figure 16) [83]. It is known that styrylBodipy shows an absorption in the red spectral region [30], and the triplet state energy (1.0 eV) is much lower than that of the native Bodipy (ca. 1.61 eV) [73,84].

**Figure 17:**
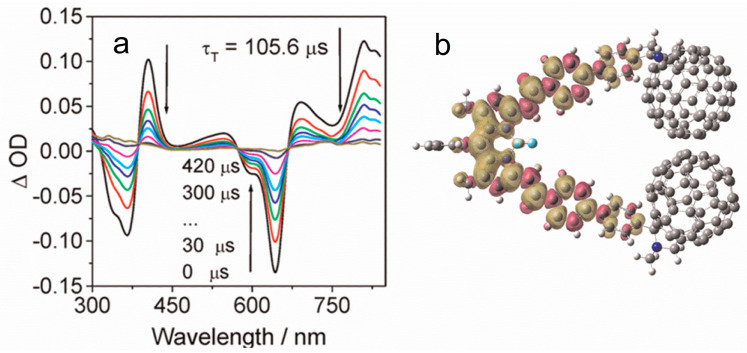
(**a**) Nanosecond transient absorption spectra of styrylBDP-C_60_-1 (color lines: ns-TA spectra at different time). In deaerated toluene, *λ*_ex_ = 532 nm, 20 °C. (**b**) Spin density surface of the T_1_ state of the styrylBDP-C_60_-1. Calculated at the B3LYP/6-31G(d) level with Gaussian 09. Reproduced with permission from reference [83], copyright 2012, American Chemical Society.

The three styrylBodipy-C_60_ dyads (styrylBDP-C_60_-1, styrylBDP-C_60_-2 and styrylBDP-C_60_-3) show a strong absorption band at ca. 640 nm, and the molar absorption coefficients are up to 65,000 M^−1^ cm^−1^. The fluorescence of the antenna is strongly quenched in the dyads (Φ_F_ are less than 1%). In comparison, the native antenna shows a Φ_F_ of 60%. A drastically different ns-TA spectral character was observed for styrylBDP-C_60_-1 as compared to C_60_ (Figure 17). Positive absorption bands in the range of 370–450 nm, 450–700 nm, and beyond 750 nm were observed, which are not characteristic ESA bands of the ^3^C_60_ state, but are, rather, the absorption of the ^3^styrylBodipy state [85]. Thus, we conclude that the T_1_ state of the dyad is localized on the styrylBodipy unit in the dyad. This is supported by the strong GSB band at 650 nm and 370 nm. The triplet state lifetime was determined as 105 μs. This lifetime is much longer than the triplet state lifetime of the styrylBodipy chromophore accessed with the heavy atom effect (ca. 2 μs) [85,86]. We confirmed that these Bodipy- and styrlBodipy-C_60_ dyads are efficient photocatalysts for photoredox organic reactions [87,88].

### 4.3. Heavy Atom-Free Triplet PSs Based on Rhodamine-C_60_ Dyads

**Figure 18:**
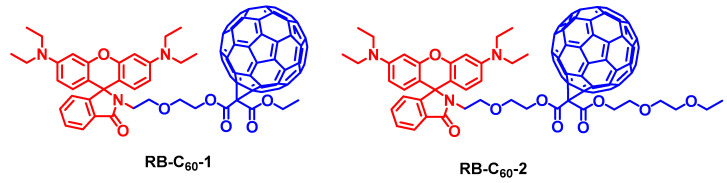
Rhodamine-C_60_ dyads showing acid-activatable ^1^O_2_ production capability. Only the opened form shows a strong absorption of visible light.

In order to endow the visible light-harvesting antenna-C_60_ dyads with more functionalities, such as an external stimuli responsive character (acid, base, etc.), which is important for the development of targetable/activatable PDT reagents [4], we prepared rhodamine-C_60_ dyad (RB-C_60_-1 and RB-C_60_-2), shown in Figure 18 [89]. The rationales of designing such molecules are that the rhodamine moiety is acid-/base-responsive. In acidic conditions, the rhodamine will be in opened form, giving a strong absorption of visible light. The absorption band is centered at 550 nm, and the molecular absorption coefficient is up to 1.1 × 10^5^ M^−1^ cm^−1^. In the presence of base, however, the rhodamine units adopts the lactam structure (the closed form), which gives no absorption in the visible spectral region [90]. Thus, we envisage that the ^1^O_2_ photosensitizing ability of the dyads can be turned on in the presence of acid, and turned off in the presence of base. We found that the Φ_Δ_ of the dyads is negligible in the absence of acid (excited at ca. 550 nm), and Φ_Δ_ is up to 88.5% in the presence of acid. RB-C_60_-1 and RB-C_60_-2 were applied in PDT for HeLa cells with a concentration of 15 μM. This is particularly of interest because targeted PDT can be achieved with such acid-responsive triplet PSs [4,91].

**Figure 19:**
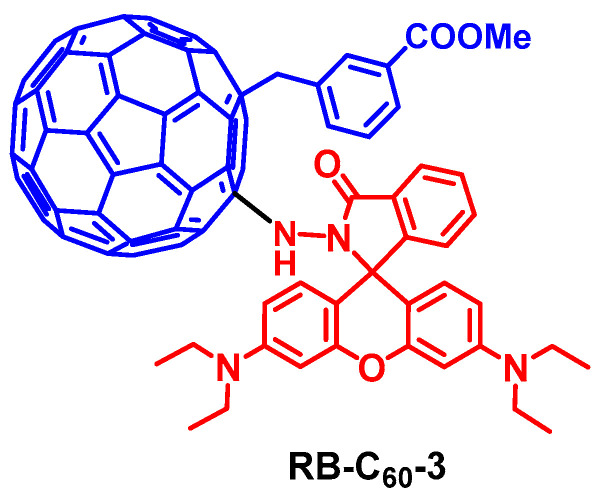
Rhodamine-C_60_ dyad showing acid-activatable ^1^O_2_ production capability, used for the PDT study.

Dong et al. undertook a detail investigation into the applicability of a rhodamine-C_60_ dyad as an acid-activatable PDT reagent (RB-C_60_-3, Figure 19) [92]. RB-C_60_-3 shows an outstanding singlet oxygen generation yield (Φ_Δ_ = 0.95) in acidic conditions, which was much higher than that under neutral conditions (Φ_Δ_ = 0.25). The Φ_Δ_ of pH-activated RB-C_60_-3 was higher than some FDA-approved photodynamic drugs (such as Photofrin^®^, Φ_Δ_ = 0.83). Through encapsulation with amphiphilic DSPE-mPEG2000, water-soluble nanoparticles (NPs) containing RB-C_60_-3 were obtained. In vitro experiments indicate that RB-C_60_-3 NPs were capable of cellular uptake and lysosomal activation (pH 4.5–5.0), and an excellent photodynamic therapeutic effect was observed (IC_50_ = 63 μM for the HCT-116 cells). The native rhodamine chromophore was applied in PDT for the T47D cell with an IC_50_ value of 5.2 μM [93]. The efficient ^1^O_2_ generation enabled by pH-activated RB-C_60_-3, or similar dyads, constitutes a novel paradigm molecular structure of theranostic reagents for cancer treatment. A supramolecular approach to combine the functionality of the antenna and the C_60_ unit is found to also be effective [94]. We confirmed this versatile strategy by using other visible light-harvesting chromophores, such as PBI [95], and NDI [96]. We also demonstrated that these compounds can be used as efficient photocatalysts for photoredox catalytic organic reactions, such as the aerobic oxidation of aromatic boronic acids and the photooxidation of 1,5-dihydroxynaphthalene [87,95].

### 4.4. Heavy Atom-Free Triplet PSs Based on PyridoneBodipy-C_60_ Dyads

**Figure 20:**
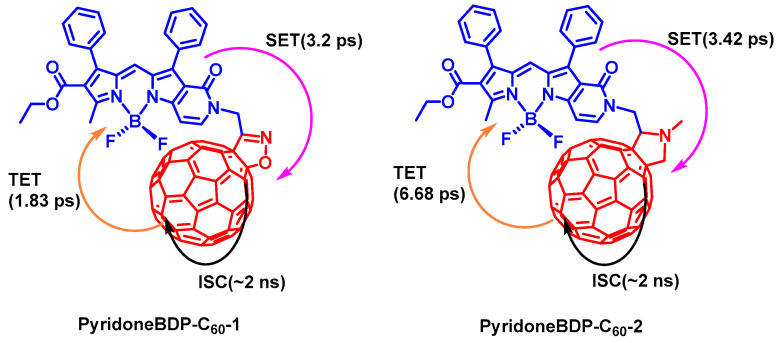
PyridoneBodipy-C_60_ dyads showing ping-pong energy transfer, i.e., the forward singlet energy transfer from pyridoneBodipy unit to the C_60_ unit, ISC of the C_60_ unit, and, subsequently, the backward triplet energy transfer from the C_60_ unit to the Bodipy unit in the dyads. The excited state kinetics are presented.

Recently, Blank et al. studied the singlet and triplet energy transfer kinetics in Bodipy-C_60_ dyads using fs-TA spectroscopy (Figure 20) [97]. Two pyridoneBodipy−fullerene dyads (compound PyridoneBDP-C_60_-1 with an isoxazole bridge, and compound PyridoneBDP-C_60_-2 with an *N*-methylpyrrolidine bridge) were investigated. Excitation is initially localized on the pyridoneBodipy unit, then singlet state energy transfer (SET) to the fullerene unit occurs (takes ca. 3 ps), the subsequent ISC of the C_60_ unit takes ca. 2 ns. Subsequently, backward triplet state energy transfer (TET) to the pyridoneBodipy occurs. This ping-pong energy transfer mechanism resulted in an efficient (>85%) overall conversion of the photoexcitation into the triplet state. The *N*-methylpyrrolidine bridge slows the backward TET (ca. 6 ns) as compared to that of dyad PyridoneBDP-C_60_-1 (ca. 1.8 ns). The triplet state lifetimes of the dyads PyridoneBDP-C_60_-1 and PyridoneBDP-C_60_-2 were determined as 1.0 μs and 1.66 μs in deaerated solution, respectively.

## 5. Intersystem Crossing of Organic Compounds Have Energy-Matched S_1_/T_n_ States

### 5.1. Energy-Matched S_1_/T_n_ States of Naphthalimide Derivatives

The lack of ISC capability in most aromatic compounds having a planar molecular structure is the large *J*, resulting from the significant overlap of the HOMO and LUMO orbitals, and the large S_1_/T_1_ states energy gap (2*J*) [10]. According to the Fermi golden rule, S_1_/T_1_ states with a small energy gap are advantageous for efficient ISC. The upper triplet excited state may exhibit a similar energy to the S_1_ state, although it is difficult to predict the energy of the upper triplet state, such as the T_2_ state. However, recent examples infer that the energy matching of the singlet excited state and the upper triplet state are more common than usually believed, and it may play a general role for enhancing the ISC [72,98,99]. This strategy may witness more development in the future. Herein, we briefly discuss several recent examples.

**Figure 21:**
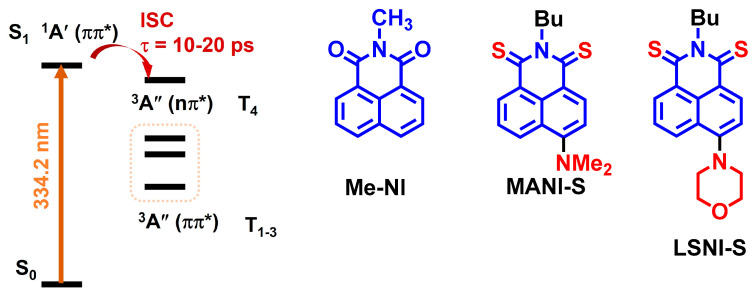
Molecular structure of *N*-methyl-1,8-naphthalimide (Me-NI) and the ISC mechanism. The derivatives of Me-NI show efficient PDT application (MANI-S and LSNI-S). Reproduced with permission from reference [100], copyright 2016, American Chemical Society.

A study of the excited state dynamics of *N*-methyl-1,8-naphthalimide (Me-NI) (Figure 21), a popular chromophore in photochemistry, in the gas phase by picosecond time- and frequency-resolved multiphoton ionization spectroscopy, aided by theoretical computation, shows that the ISC of NI occurs between the S_1_ and T_4_ states [100]. These two states share similar energies and the electronic configurations of the two states are different; thus, El Sayed’s rule for ISC is satisfied (Figure 21). The ISC takes 10–20 ps, which can explain the weak fluorescence of the NI chromophore [101]. However, the native NI chromophore only shows absorption in the spectral range of 300–360 nm, which is clearly not suitable to be used as a visible light excitable triplet PSs for application in PDT [102]. Although amino substitution, for instance, at the 4-position of the NI core, can red-shift the absorption wavelength, the ISC vanishes upon this kind of derivatization [103,104,105]. Other examples also show that the delicate energy matching between the S_1_ state and the T_n_ state can be easily broken by subtle modification of the chromophore, for instance, by attaching a phenyl moiety on the 4-position of the NI moiety, we found that the 4-pheylNI shows a Φ_F_ of 95%, meaning that the ISC is greatly reduced [102].

With replacing the oxygen atom of the carbonyl groups in NI chromophore with sulfur atoms, the resulting thiocarbonyl naphthalimide (MANI-S) (Figure 21) shows efficient formation of triplet state upon photoexcitation, which is due to the small energy gap between the S_1_ and T_3_ states [21]. MANI-S shows an excellent PDT effect, even under a severely hypoxic environment (1% O_2_), which was superior to that of the clinical PDT regent MB. The photochemical reaction in water and biomacromolecules (10% fetal bovine serum) was studied for LSNI-S [106], which demonstrated that the superoxide (O_2_^∙−^) is produced through electron transfer from the triplet excited state of PS to the proximate substrates.

### 5.2. Energy-Matched S_1_/T_n_ States of Naphthalenediimide Derivatives

**Figure 22:**
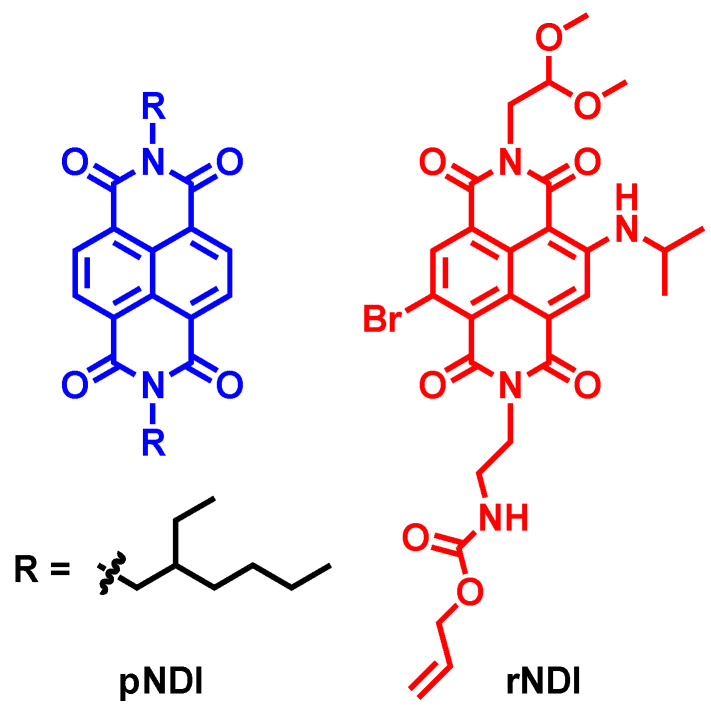
Molecular structures of the NDI compounds rNDI and pNDI.

**Figure 23:**
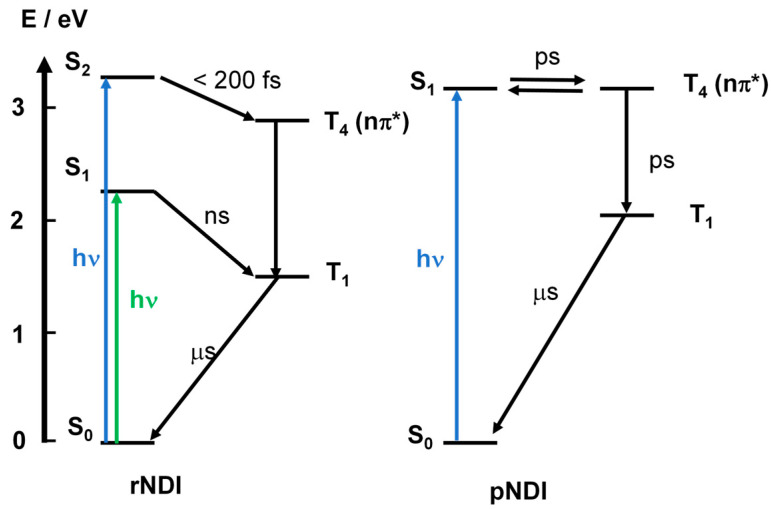
ISC mechanisms of the compounds rNDI and pNDI. Reproduced with permission from reference [110], copyright 2015, American Chemical Society.

Similarly, the plain naphthalenediimide (pNDI. Figure 22) shows ISC between the S_1_ and T_4_ states. Using fs-TA spectral characterization, global fitting, and target analysis of the data, the T_4_ state can be identified (Figure 23), since the spectra character is different from the T_1_ state, which can be feasibly confirmed by ns-TA spectra [107]. It should be noted that the upper triplet state of a chromophore is usually difficult to be detected, due to ultrafast internal conversion (IC) to the T_1_ state [108]. For rNDI, however, with the amino group introduced to the NDI core, the S_1_ state energy decreases (the absorption wavelength is red-shifted to 530 nm, for pNDI, the maximal absorption wavelength is ca. 370 nm). As a result, the S_1_/T_4_ states energy matching is changed; fast ISC occurs from the S_2_ to the T_n_ state, whereas the S_1_→T_1_ ISC is much slower (on the nanosecond-scale), because of the large energy gap. The ISC capability of rNDI is due to the heavy atom effect of the bromine atom attached on the NDI core. We found that without the Br atom attached, the amino-substituted NDI shows poor ISC [41,44,96,109,110].

### 5.3. Energy-Matched S_1_/T_n_ States of Perylenebisimide Derivatives

**Figure 24:**
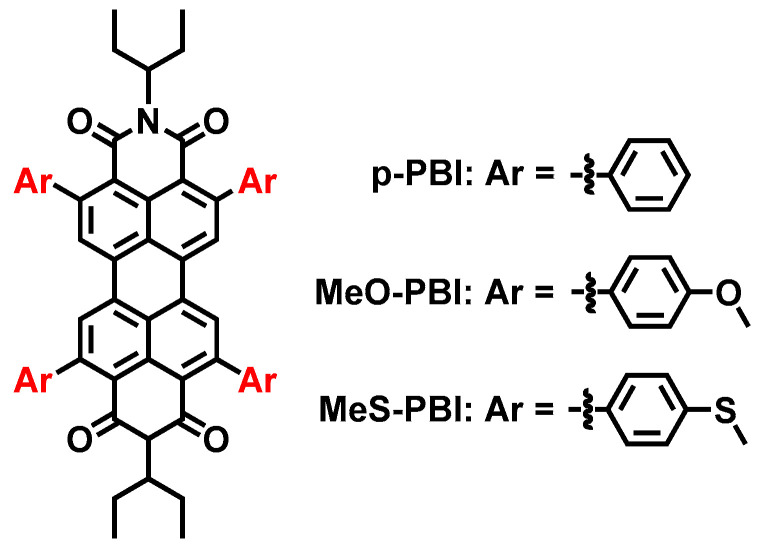
Heavy atom-free PBI derivatives showing ISC.

Concerning visible light-harvesting ability, PBI is of particular interest [111,112]; however, the native PBI only shows strong fluorescence and the ISC is negligible [113,114,115]. Fu et al. reported a series of PBI derivatives with substituents attached at 2,5,8,11-positions, whereby a decent ISC was observed for the derivatives with electron-donating groups attached to the phenyl ring (Figure 24). For instance, MeO-PBI shows a Φ_Δ_ of 50%, and MeS-PBI shows a Φ_Δ_ of 80% [116]. These compounds show strong absorption at ca. 530 nm. fs-TA spectra show that the ISC of MeO-PBI takes ca. 28 ps, and the ISC of MeS-PBI takes about 84 ps. The triplet state lifetimes of the two compounds were determined in deaerated solution as 59.7 μs and 61.4 μs, respectively, by ns-TA spectra. It was proposed that the ISC occurs between the S_1_/S_n_ states and the T_n_ state. Direct ISC to the T_1_ state is believed to be weak due to the large energy gap of S_1_/T_1_ states.

**Figure 25:**
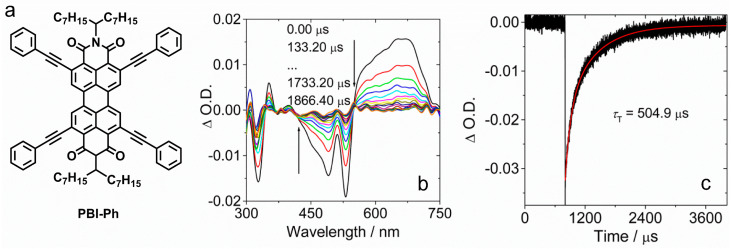
(**a**) Molecular structure of PBI-Ph. (**b**) Nanosecond transient absorption spectra of PBI-Ph. (c) Decay curves of PBI-Ph at 532 nm (black line: experimental spectrum, red line: fitting result). *λ*_ex_ = 530 nm, *c* = 1.0 × 10^−5^ M in deaerated toluene; 20 °C. Reproduced with permission from reference [117], copyright 2016, American Chemical Society.

Independently, we found that the PBI derivative with 2,5,8,11-tetraphenylacetylene substituents PBI-Ph also shows efficient ISC (Figure 25) [117]. The compound shows a strong absorption of green light, and the molar absorption coefficient is up to 73,300 M^−1^ cm^−1^ at 532 nm. However, the Φ_F_ of this compound is only 7%, which is much lower than the native PBI (ca. 86%) [52,118], and the derivative with two phenylacetylene substituents attached at the 1,7-positions (bay position. Φ_F_ = ca. 90%). The Φ_Δ_ of the compound was determined as 51-66% in different solvents. The ns-TA spectra of the compound show a broad ESA band in the range of 530–750 nm. This character is different from the ^3^PBI state, which shows a broad ESA band in the range of 400–600 nm [119]. The triplet state lifetime was determined as 505 μs in deaerated solution. Note that this triplet state lifetime is much longer than the PBI triplet state accessed with the heavy atom effect of Br (0.41 μs) [47,117,120], REISC (1.5 μs) [47], or in cyclometalated Ir(III) complexes (22 μs) (PBI is remote from the Ir(III) coordination center) [121], or in N^N Pt(II) bisacetylide complexes in which the PBI is attached to the Pt(II) center via an acetylide bridge (0.246 μs) [122].

Our results demonstrated the advantage of the heavy atom-free triplet PSs, i.e., the long-lived triplet state, as compared to that accessed by the heavy atom effect. It should be pointed out that the matching of the upper singlet and triplet excited states is more common than usually thought, and the strategy has been exploited in other areas, such as the hot exciton approach in the design of efficient electroluminescence materials [123]. This strategy may witness further development in triplet PSs used for PDT and photocatalytic synthetic organic reactions, or organic photovoltaics.

## 6. Concluding Remarks

In summary, we briefly discussed the recent developments of heavy atom-free organic triplet photosensitizers (PSs) that can be used for photodynamic therapy (PDT). This topic is interesting with regard to both fundamental photochemical and PDT studies, because without heavy atoms, the intersystem crossing (ISC) of organic compounds becomes elusive and difficult to predict. Thus, strategies to achieve efficient ISC in organic compounds are interesting, but only rules with limited validity are known. On the other hand, heavy atom-free triplet PSs have advantages of a low dark toxicity, a low cost of preparation, and longer triplet state lifetimes, compared to their counterparts containing heavy atoms, such as Ir, Pt, Ru, or Br and I atoms. Moreover, the new heavy atom-free organic triplet PSs can undergo further derivatization; thus, modification of the molecular structure for targeting PDT is possible, which is different to porphyrin/porphine-like PDT reagents. In this review, we discussed the recent developments of radical-enhanced ISC (REISC), twisted π-conjugation system-induced ISC, the use of fullerene C_60_ as an electron spin converter in antenna-C_60_ dyads, and, finally, energy-matched S_1_/T_n_ states-enhanced ISC. Most of the examples introduced in this review are from our group. These topics are interesting in fundamental photochemistry studies, and the related compounds have been demonstrated as promising PDT reagents. We anticipate more developments in these areas in the near future.

## Data Availability

Not applicable.
